# AI passing and invisible authenticity labor: trust vulnerabilities and inequality in China's short-video creator economy

**DOI:** 10.3389/fpsyg.2026.1800866

**Published:** 2026-04-28

**Authors:** Xiaolong Su, Tingli Liu, Zhuo Ye, Yixue Mei

**Affiliations:** 1 State Key Laboratory of Media Convergence and Communication, Communication University of China, Beijing, China; 2 School of Journalism, Communication University of China, Beijing, China

**Keywords:** AI passing, authenticity labor, generative AI, invisible work, short-video platforms, Xiaohongshu

## Abstract

Generative AI is increasingly embedded in China's short-video production, yet on authenticity-oriented platforms its visible use can be discrediting. Drawing on 16 in-depth interviews with creators active on Xiaohongshu and Douyin, this article introduces AI passing: creators' strategic efforts to conceal and humanize AI-assisted drafts so that outputs plausibly appear human-authored. We show that creators associate legible AI assistance with intertwined trust vulnerabilities, including epistemic unreliability, anticipated relational penalties, and platform authenticity regimes. To manage this legibility, creators perform four recurring forms of invisible authenticity labor: epistemic verification, linguistic naturalization, narrative restructuring, and performative embodiment. These practices reallocate work from generation to downstream repair and performance, complicating claims that AI simply improves efficiency. Finally, we demonstrate that passing capacity is stratified by educational and professional capital, economic resources and team support, and platform position, producing trust-based inequality in who can leverage AI while sustaining credibility, voice, and liveness.

## 1 Introduction

Generative AI has rapidly entered everyday media production, reshaping how digital content is imagined, drafted, and circulated. In China's short-video economies, creators increasingly rely on AI for ideation, scripting, captions, and optimisation—often as a practical response to relentless rhythms of posting and platform competition. Yet in spaces where creators are expected to appear spontaneous, personal, and “real,” AI assistance can become a liability rather than an asset: something to be used, but not seen. A Douyin creator (S1) captured this tension vividly. Although an AI tool can draft her script within minutes, she spends far longer rewriting it “to sound like me”—removing formal phrases, inserting

habitual catchphrases, and even planning small “mistakes” to simulate spontaneity. Asked why she does not disclose AI assistance, she replied that once refined, the output should be “untraceable.” If generative AI is widely framed as an efficiency tool, why does it so often generate additional work aimed at making its involvement disappear?

This puzzle sits at the intersection of platform authenticity norms and algorithmically mediated authorship. Creator and influencer studies show that “authenticity” is produced through sustained backstage labor: crafting intimacy, ordinariness, and a recognizable personal voice while keeping the work of production out of sight (Abidin, 2016, 2021; Duffy, 2017). Visibility and monetisation are further shaped by platform governance and algorithmic curation, creating incentives to adapt content and self-presentation to opaque systems of ranking and recommendation (Cotter, 2019). In others, visible algorithmic involvement can be penalized—particularly after observed errors (Dietvorst et al., 2015)—even as people may prefer algorithmic judgement under certain conditions (Logg et al., 2019). Taken together, existing research suggests that trust in algorithmically mediated production depends on norms and on how mediation becomes legible (Dietvorst et al., 2015; Logg et al., 2019). Less understood, however, is how creators operationally manage that legibility when AI is embedded in everyday writing and scripting workflows where authenticity is a central evaluative frame.

China's short-video platforms provide a revealing site for examining these dynamics. Platforms such as Xiaohongshu and Douyin valorise “genuine experience,” relatable ordinariness, and a consistent personal voice. In such environments, visible AI assistance may be read as misaligned with sincerity or authorship—not necessarily because audiences reject technology *per se*, but because they evaluate content through an authenticity lens. Disclosure thus becomes a complex strategy: transparency can shape trust, but its effects are not straightforward and depend on how information is presented and interpreted (Kizilcec, 2016). For creators whose value depends on a recognizable persona, a plausible response is not simply to avoid AI, but to use it while actively managing its traceability.

To theorize this practice, this article introduces AI passing: the strategic effort to conceal and humanize AI-assisted content so that it plausibly passes as fully human-authored. Building from Goffman (1963) account of “passing” as the management of potentially discreditable information, we conceptualize AI passing as a sociotechnical practice rather than a stable identity trait. AI passing is not reducible to non-disclosure; it involves concrete labor aimed at removing recognizable “AI traces” and restoring personal voice, experiential plausibility, and embodied presence. Analytically, we treat AI passing as a form of situated risk management under authenticity expectations, rather than as a claim about creators' intentions or morality. When successful, this work becomes doubly invisible: audiences encounter “natural” content, while both the AI's role and the human labor required to domesticate it remain hidden.

Drawing on 16 in-depth interviews with creators operating across Chinese platforms (primarily Xiaohongshu, with substantial Douyin presence) and routinely using AI tools for text-based tasks (e.g., scripting and copywriting), this article addresses two research questions: What trust- and authenticity-related risks do creators associate with visible AI assistance in short-video production? What forms of invisible authenticity labor constitute AI passing in creators' AI-assisted workflows?

This study makes three contributions. First, it offers AI passing as a transferable concept for analyzing how creators manage the legibility of AI assistance in authenticity-oriented creator economies. Second, it specifies the labor of passing by identifying four recurring forms of invisible authenticity work: epistemic verification, linguistic naturalization, narrative restructuring, and performative embodiment. Third, it advances debates on AI and creative work by showing how generative AI can relocate labor into less visible, more skill-intensive forms of repair and performance, work that is central to sustaining authenticity and trust in short-video production. The remainder of the article proceeds as follows. The next section reviews scholarship on platformed authenticity and trust in algorithmically mediated production. We then outline the study's methodology, before presenting findings on four forms of invisible authenticity labor that constitute AI passing. The conclusion discusses implications for research on creator economies, trust, and AI-enabled cultural production.

## 2 Literature review

### 2.1 Authenticity regimes and visibility labor in platformed cultural production

Authenticity is widely treated as a key currency in creator economies. While psychological research has treated authenticity as a multidimensional individual disposition (Kernis and Goldman, 2006), research on platform economies consistently shows that authenticity is not a natural attribute that creators either possess or lack (Abidin, 2016, 2021; Duffy, 2017). Rather, authenticity is produced through labor and craft—a dynamic more akin to Goffman (1959) dramaturgical account of self-presentation than to any straightforward expression of inner truth. This includes the ongoing calibration of voice, intimacy, and “everydayness” in ways that remain credible to audiences. Abidin (2016) conceptualizes this as visibility labor, namely the sustained work of making oneself and one's content perceptible and valuable within platform environments while keeping the work itself unobtrusive. Duffy (2017) similarly demonstrates how aspirational labor normalizes intensive emotional and entrepreneurial effort as passion, obscuring the structural pressures that shape who can persist in highly competitive attention markets.

These pressures are not only cultural but also infrastructural. Platform governance and algorithmic curation shape what becomes visible, which creator practices are rewarded, and how quickly trends and genres stabilize. Cotter's (2019) study of Instagram shows how influencers strategically negotiate with algorithmic systems, developing tactics to manage reach, relevance, and perceived authenticity under conditions of uncertainty. Chinese scholarship on short-video and influencer cultures adds specificity to how authenticity norms and platform governance co-evolve in local settings, including the moral language used to evaluate “realness,” the importance of experiential credibility, and the practical consequences of being perceived as “performative” or overly commercial on platforms such as Douyin and Xiaohongshu (Ding and Xing, 2023; Lin and Ruan, 2024). Related Chinese-language research also points to the routinisation of optimisation practices and the increasing standardization of creator workflows under recommendation systems, which can intensify the need to present “natural” content while actively engineering its circulation (Chen and Zhang, 2023; Yu and Teng, 2023).

Taken together, this work clarifies why authenticity is both valuable and costly in platformed production. It also establishes a concrete expectation: when creators adopt generative AI, they are not simply adding a tool to a neutral workflow but introducing a potentially controversial mediator into an already moralized economy of authenticity. Before we can theorize how creators manage this mediation, however, we need to understand what shapes audience evaluations of algorithmic involvement—a question to which we now turn.

### 2.2 AI assistance, audience inference, and the conditionality of trust

Work on human responses to algorithms suggests that audience evaluation of algorithmic mediation is contingent rather than uniform. Understanding this contingency requires a clear account of what trust entails. Organizational psychologists have long argued that trust involves assessments of another party's ability, benevolence, and integrity (Mayer et al., 1995), a framework that has proven robust across disciplines and contexts (Rousseau et al., 1998). In the context of algorithmically mediated cultural production, these dimensions translate into whether audiences perceive a creator, and the tools they use, as competent, well-intentioned, and honest about their creative process.

Experimental research demonstrates that such assessments are far from automatic. Dietvorst et al. (2015) show algorithm aversion, where people become reluctant to rely on an algorithm after seeing it make mistakes, even when the algorithm performs well overall. At the same time, Logg et al. (2019) document algorithm appreciation, where people in many judgement contexts prefer algorithmic advice to human advice. Critically, the direction of these evaluations appears to depend on task domain: people are more resistant to algorithmic input for tasks perceived as subjective or requiring human sensibility than for those perceived as objective (Castelo et al., 2019), a finding with direct implications for creative content production. These findings are particularly useful for creator economies because it discourages a simple prediction that AI involvement automatically harms perceived quality or credibility, while also suggesting that creative and expressive domains may be especially sensitive to perceived algorithmic mediation.

More recent studies further highlight that algorithm use can shape evaluations of the producer, not only the output. Jago and Carroll (2023) show that people vary in the degree to which they credit humans vs. algorithms, which complicates how “originality,” “effort,” and “authorship” are inferred. Gondwe (2025) finds that trust in AI-generated news across ten African countries is generally neutral rather than categorically negative, with significant variation by age and digital literacy. Lee and Cha (2025) demonstrate that the relationship between AI transparency and trust is dynamic rather than linear, with transparency dimensions such as accuracy and clarity influencing trust only when they interact with perceived functionality and helpfulness. These findings collectively suggest that audience trust in AI-mediated content is not a fixed disposition but a situated judgement shaped by what the AI is perceived to do, how legibly it does it, and whether the outcome meets contextual expectations. For short-video cultures that center personality and voice, these dynamics are especially consequential, because perceived authorship is often inseparable from creator persona.

Debates about transparency provide a further hinge between technical mediation and trust. Kizilcec (2016) finds that the amount and form of information provided about algorithmic systems affects trust, but not in a straightforward more-is-better manner. Henestrosa and Kimmerle (2025) extend this insight to the domain of generative AI, showing across three experimental studies that disclaimers about AI authorship fail to univocally influence the perception of AI-generated content: disclaimer type did not affect message credibility, and its effects on source credibility were inconsistent and not always in the expected direction. Notably, evaluative information about AI's capabilities lowered credibility perceptions in some conditions, suggesting that disclosure can backfire when it makes the limitations of the tool salient without offering audiences a clear interpretive frame.

Chinese research on platform governance and user perceptions adds that transparency is interpreted through local expectations about platform power, monetisation, and manipulation. Disclosure can be read as accountability in some contexts, and as strategic performance in others, which means transparency does not automatically settle authenticity disputes (Pan and Liu, 2025; Lu, 2025; Chen and Qiu, 2023). For creators, the implication is practical. If audiences treat visible AI use as a cue for low sincerity or low effort, then openly disclosing AI assistance can become a reputational risk rather than an ethical safeguard. This literature explains why creators face uncertain payoffs when they reveal or conceal AI involvement. What it does not yet specify is the work required to make AI assistance socially legible, or to make it socially ignorable. That missing link motivates our focus on passing practices.

### 2.3 AI passing as invisible authenticity labor: passing, repair, and maintenance

To address this gap, we propose AI passing as a concept for analyzing how creators manage the legibility of AI assistance under authenticity regimes. The concept builds on Goffman's dramaturgical account of self-presentation (1959) and, more specifically, his theorisation of passing as the management of potentially discreditable information (1963). Importantly, passing as an analytic framework has already been generalized beyond its original context of stigmatized social identities. DeJordy (2008) repositions passing as a structural feature of any organizational situation in which an individual possesses an invisible attribute perceived as discreditable, detaching the concept from specific identity categories and thereby authorizing its migration into new empirical domains—including, as we argue here, AI-assisted cultural production, where the use of generative tools can itself function as a potentially discreditable attribute under prevailing authenticity norms. The concept also draws on the psychological distinction between impression motivation, the perceived need to control how one is perceived, and impression construction, the specific strategies through which desired images are enacted (Leary and Kowalski, 1990). In the case of AI-assisted creation, creators are motivated to manage impressions because visible AI use may threaten perceived authenticity; the specific practices they deploy, such as rewriting, re-voicing, and selective disclosure, constitute their impression construction strategies. Importantly, the analytic point is not that creators always deceive audiences, but that they operate in contexts where visible AI assistance may be interpreted as a discrediting cue, requiring strategic management of information, performance, and voice.

AI passing can also be understood through scholarship on invisible work, repair, and coordination. Star and Strauss (1999) show that essential work often becomes invisible when it succeeds, creating “layers of silence” around the labor that sustains everyday systems. Strauss (1985) conceptualizes articulation work as the connective tissue of practice, involving the coordination, adjustment, and alignment that enables tasks to be completed under real-world constraints. From a broader perspective, infrastructures and sociotechnical arrangements persist through ongoing maintenance rather than seamless operation (Graham and Thrift, 2007). Jackson (2014) further argues that repair is not merely a response to breakdown but a generative practice through which sociotechnical worlds are continually remade. Applied to generative AI, these perspectives reveal that what appears as automation is often enabled by human repair: checking, rewriting, contextualizing, and re-performing machine outputs so they fit situational norms. Recent empirical work on human—AI co-creation lends further support for this view (Wang et al., 2025; Gonzalez et al., 2024), as discussed in detail below.

In authenticity-oriented short-video production, repair takes a specific form. Creators must transform AI drafts into a recognizable personal voice and an embodied performance that can sustain audience trust. This aligns with Gonzalez et al. (2024) argument that in AI-saturated organizational contexts, the “human touch” can become more, not less, central to craft and legitimacy. Wang et al. (2025) provide complementary empirical evidence, showing that while a structured co-creative process improved designers' creative outcomes in character concept design, AI-generated materials still required deliberate filtering, recombination, and stylistic refinement before they could constitute viable work. Although their setting of character concept design differs from short-video production, the underlying dynamic is transferable: generative AI shifts rather than eliminates creative labor, redirecting it toward the evaluative and performative work through which machine outputs are rendered situationally appropriate. Gonzalez et al.'s emphasis on the value placed on human presence and craft, together with Wang et al.'s evidence that co-creative output depends on sustained human judgment, provides a dual lens for understanding why creators might invest labor in making AI assistance less visible and more socially acceptable.

However, existing research has not yet specified how these dynamics are operationalised in everyday creator workflows. We lack a detailed account of the concrete practices through which creators transform AI-assisted drafts into content that can pass as human-authored, and we know little about how the resources required for such practices are distributed across differently positioned creators. This study addresses both gaps by empirically tracing AI passing as a form of invisible authenticity labor within China's short-video creator economy, examining the specific work it entails and the inequalities it produces.

## 3 Methodology

### 3.1 Research design and sampling strategy

This article adopts a qualitative interview design to examine AI passing, defined as creators' efforts to conceal, humanize, or re-voice AI-assisted drafts so that outputs plausibly appear human-authored under platform authenticity expectations. We conducted 16 semi-structured interviews with short-video creators in China between June and September 2025.

We used purposive sampling, supplemented by snowball sampling, to recruit participants for whom both authenticity norms and AI-assisted writing were practically consequential. Recruitment occurred through creator networks and platform-based outreach. Eligibility criteria were: (1) active posting on at least one major short-video platform; (2) routine use of generative AI tools for text-based tasks such as scripting, copywriting, titles, and captions; and (3) engagement in persona- and voice-centered content where authenticity norms were salient. We sought variation across niches, creator trajectories, and follower scales in order to identify shared mechanisms as well as meaningful differences in how AI passing is enacted.

Participants operated across multiple platforms, primarily Xiaohongshu (10/16), with substantial Douyin presence (5/16), and additional activity on Kuaishou (2/16), Bilibili (2/16), and WeChat (3/16). The sample spans ages 18–44 (13 women and 3 men) and includes follower counts ranging from hundreds to over 300,000, enabling comparison across long-tail and more established creators. The study is designed for mechanism-building rather than platform comparison; platform differences are treated as contextual conditions that shape how AI passing is performed (see Table 1).

**Table 1:** Participant demographics and platform distribution.

ID	Gender	Age	Platform(s)	Category/niche	Occupation	Education	Update frequency	Followers
S1	Female	36	Douyin	News commentary	Host	Bachelor's	Every 3 days (avg.)	153k
S2	Female	26	Xiaohongshu, Bilibili	Comedy	Internet operations	Bachelor's	1–2 posts/week	1.2k
S3	Male	22	Kuaishou	Travel vlog	Student	Bachelor's	Weekly	24k
S4	Female	30	Xiaohongshu	Knowledge and culture	Editor	Master's	Weekly	157k
S5	Female	29	Kuaishou, Douyin, WeChat	Cultural tourism promotion	Converged media ops	Master's	Daily	306k
S6	Female	44	Douyin, WeChat	Traditional culture	Teacher	Master's	Weekly	1.2k
S7	Male	33	Xiaohongshu	Lifestyle, knowledge	University teacher	Ph.D.	Every 10 days (avg.)	25k
S8	Female	23	Xiaohongshu	Life sharing	Student	Master's	Weekly	2.7k
S9	Female	25	Xiaohongshu	Life sharing	Student	Master's	Weekly	284
S10	Female	26	Xiaohongshu	Life sharing	Student	Bachelor's	Every 10 days (avg.)	1,469
S11	Female	21	Xiaohongshu	Life sharing	University counselor	Master's	Twice a week (avg.)	190
S12	Male	18	Xiaohongshu, Bilibili	Campus vlog	Student	Bachelor's	Twice a week (avg.)	10k
S13	Female	18	Xiaohongshu, WeChat	Self-made variety show, life sharing	Student	Bachelor's	Weekly	92k
S14	Female	25	Douyin	Beauty and fashion	Programmer	Bachelor's	3 times/week	1.1k
S15	Female	24	Xiaohongshu, Douyin	Language knowledge	Advertising operations	Bachelor's	3 times/week	1.8k
S16	Female	20	Xiaohongshu	Opinion and knowledge	Student	Bachelor's	Daily	512

### 3.2 Data collection

Interviews were conducted online in Mandarin and audio-recorded with participants' consent. We followed a semi-structured guide covering production routines, recent episodes of AI-assisted writing, perceptions of authenticity cues, and decisions around disclosure or non-disclosure of AI assistance. To reduce generic self-reporting and improve recall precision, we used incident-based prompts and asked participants to walk through a recent AI-assisted post step by step, focusing on what was generated, what was revised, what sounded “AI-like,” and why particular edits mattered for credibility. Interviews lasted approximately 60–120 min. Recordings were transcribed verbatim and de-identified during transcription. Participants are referred to using pseudonyms (e.g., S1).

### 3.3 Analysis and ethics

Transcripts were analyzed iteratively using thematic coding. We combined sensitizing concepts from scholarship on authenticity labor and invisible work with inductive attention to participants' categories and routines. First-cycle coding captured concrete practices used to transform AI outputs, such as verifying claims, rewriting phrasing to match personal voice, restructuring story progression, and adjusting text to support a more embodied performance. Second-cycle coding grouped these practices by function in sustaining credibility under authenticity expectations. This process yielded four recurring forms of invisible authenticity labor that constitute AI passing across the sample: epistemic verification, linguistic naturalization, narrative restructuring, and performative embodiment. We used constant comparison across interviews to identify shared mechanisms as well as variation across niches, follower scales, and platform contexts, including cases where participants reported partial disclosure or framed AI use more positively.

Given the reputational sensitivity of AI use and disclosure in creator economies, confidentiality was prioritized. Participants provided informed consent and could decline any question. Identifying details, including handles, locations, and brand references, were removed or masked during transcription and reporting. Data were stored securely, and published excerpts were selected to avoid uniquely searchable phrasing while preserving analytic meaning. This study received ethical approval from the Research Ethics Committee of our university.

## 4 Trust vulnerabilities and moral risks of AI assistance

Creators engage in AI passing within a landscape of intertwined trust vulnerabilities that make algorithmic assistance both indispensable and stigmatized. Across interviews, these vulnerabilities cluster around epistemic unreliability, anticipated relational penalties, and platformed authenticity regimes that collectively produce a “collective trap” of concealment.

### 4.1 Epistemic unreliability and verification burdens

Epistemic distrust begins with repeated encounters with hallucinations and fabricated details. S9, a lifestyle creator, described asking AI about popular topics:

“*The answers found through AI are completely different, and the three versions provided by AI show different sources. So I don't know what's true, and their credibility is still questionable*.”

S11 recounted an instance where she requested recent social news: “*What it produced was made up—I couldn't find anything about it online at all*.” When S11 asked AI for trending memes, she explained:

“*I definitely need to check online to see what it really is, where it originated, how many people are talking about the topic now, and whether the meme actually exists… This is quite important, so I'll search online—and that's when I find out it's a flop*.”

Even when facts are not obviously wrong, opacity undermines trust. S6, a cultural creator with graduate training, articulated the epistemological barrier:

“*I don't understand how AI reaches conclusions. What sources? What biases? That opacity means I can't decide when to trust it. So I verify everything, which takes forever*.”

These failures generate a basic trust paradox. Creators adopt AI to save time, yet must invest extensive labor restoring trustworthiness to AI outputs. S6 quantified the arithmetic:

“*AI generates a draft in 10 minutes. I spend 90 minutes verifying facts—checking historical records, cross-referencing scholarly sources*.”

S11 concluded that “*the time saved by AI is probably offset by the time I spend screening the ideas*.”

### 4.2 Anticipated rejection and moral/commercial stigma

Even when creators successfully repair AI's epistemic shortcomings, they anticipate audience rejection. S9 adopted an imagined viewer's stance:

“*From the audience's perspective, I also don't really want creators to generate content using AI; I would prefer them to have something with genuine feelings*.”

S12 worried:

“*Those who clicked on the video specifically to learn something useful will feel deceived when they find out it's an AI-written script or just a general template*.”

S14 evoked the emotional weight:

“*I feel like this requires some concealment… I don't have the habit of labeling, so sometimes I feel a bit guilty, like I've hired a ‘ghostwriter'*.”

Most evocatively, S16 likened AI to “dirty water”:

“*AI is like dirty water—if you tell others you used it, you can't explain whether you used it partially, reasonably, or fully. It will affect my audience's trust in my content and their recognition of my account's overall image*.”

Because audiences cannot easily calibrate “reasonable” vs. “excessive” AI use, any admission risks total contamination. Commercial relationships amplify this stigma. S15 drew a sharp line:

“*For things like scripts and copy, I think others shouldn't know—especially the merchants or clients I work with. If they find out, they will definitely be unwilling to cooperate*.”

### 4.3 Platform authenticity regimes and the collective trap

These individual anxieties are embedded in platforms' authenticity regime. Participants emphasized that platforms “prefer more authentic content” (S12), framed as grassroots sharing and “genuine user experiences.” AI becomes the wrong kind of mediation: not the invisible algorithm curating feeds, but overt algorithmic authorship contradicting the platform's self-image as a community of real-life sharers. Platform detection mechanisms reinforce concealment pressures. S15 observed:

“*Now when you scroll through the platform, sometimes it will directly prompt you that a piece of content might be AI-generated. But for me, I won't label [my own content], because I don't think the platform can identify AI traces that clearly*.”

Such algorithmic surveillance creates uncertainty about which linguistic patterns trigger detection.

Finally, creators perceive a collective trap. S15 stated:

“*Whether I continue running my account or not, I think AI can no longer be separated from humans… There is a certain degree of dependence on it*.”

Yet as AI use becomes normalized, concealment becomes the norm as well. S15 noted that if AI-generated scripts are revised, “*the platform actually can't detect it—it's similar to plagiarism*.” S16 observed that creators who refuse AI will be “at a greater disadvantage,” lacking both efficiency benefits and the ability to “go with the flow.” Individual concealment is rational: disclosure risks stigma while competitors continue to pass. Yet universal concealment erodes systemic trust: audiences suspect covert AI everywhere, while creators privately view prevailing authenticity claims as untenable. Within this context of epistemic distrust, anticipated audience rejection, and collective entrapment, AI passing emerges as a necessary condition for continued participation in the platform economy.

Not all creators, however, experienced this trap with equal force. S3, a travel vlogger on Kuaishou, described routinely disclosing his AI use:

“*Generally in two ways. Either I add a small text note in the bottom-right corner of the video, or I mention it in the rolling credits at the end. Sometimes when someone asks in the comments, I just say it directly*.”

He regarded AI-assisted scripting as “*an open secret in the industry*,” believed audiences evaluated travel content primarily on utility—“*most viewers don't seem to mind; they care more about whether the video is good and the guide is practical*”—and found that disclosure could itself become a trust asset: “*after I started labelling it, some people actually thought I was being candid—nothing to hide*.” Rather than undermining the broader pattern, S3's case sharpens its boundary conditions. His niche is oriented toward informational utility rather than the intimate, voice-driven self-expression that characterizes most other participants' genres, aligning with evidence that audiences are less resistant to algorithmic involvement in tasks perceived as objective than in those requiring human sensibility (Castelo et al., 2019). Equally telling is his framing of disclosure as persona construction: by positioning candor as a character trait, he converts a potential reputational risk into an impression management resource (Leary and Kowalski, 1990). The conditions enabling his openness—utilitarian content, output-focused audience evaluation, and a persona built around straightforwardness—are precisely the conditions most other participants reported lacking.

## 5 Invisible craft labor of making AI-generated content “authentic”

To navigate these trust vulnerabilities, creators perform intensive craft work to transform AI outputs into content that appears convincingly human. This labor is “invisible” in two senses: when effective, audiences see only “natural” posts and do not recognize either AI's role or the corrective work hiding it; when ineffective, creators bear reputational costs while the effort invested in humanizing AI remains unacknowledged. Across interviews, AI passing took the form of repair work: creators repeatedly “fix” AI outputs so that content can meet platformed expectations of credibility, personal voice, and liveness without disclosing the tool's involvement.

### 5.1 Epistemic verification

A first domain of invisible labor involves verifying and stabilizing AI-assisted claims so that creators can present content as trustworthy knowledge. Epistemic repair begins with the recognition that AI outputs can be internally inconsistent, difficult to source, or even wholly fabricated. Rather than treating AI as an authoritative reference, creators described treating it as an unstable starting point whose claims must be corroborated externally before they can be safely incorporated into scripts. For instance, when AI generates multiple incompatible versions of the same answer or points to unclear provenance, creators do not simply “choose” one; they interpret such inconsistency as a signal that the content cannot be taken at face value and must be checked against other materials (S9). Similarly, when AI produces “news-like” items that cannot be independently located, creators interpret the absence of external traces as a warning sign and discard or rewrite those segments (S11).

Creators also described verification demands that extend beyond correctness to include social existence and platform salience. For trend- and meme-based content, the question is not only whether an explanation is accurate but whether the referenced meme is actually circulating and recognizable to audiences at that moment. This requires creators to look up origins, usage contexts, and current uptake to avoid presenting “hot topics” that are, in practice, non-topics (S11). In other words, creators verify not only facts but also the cultural status of what AI proposes—whether it exists as a shared reference that can anchor attention and credibility.

Verification work is intensified by AI's opacity, which prevents creators from knowing when they can safely trust an output. S6 described this as a problem of unknowable reasoning and sourcing: without visibility into how conclusions are produced, the default stance becomes comprehensive checking. The result is that AI assistance does not simply replace labor; it reorganizes it. Drafting may become faster, but creators then absorb substantial off-screen time in checking, cross-referencing, and screening outputs before they can be presented as reliable content (S6; S11). By performing this checking invisibly, creators convert unstable AI drafts into apparently credible content—work that is central to passing precisely because it leaves no visible trace in the final post.

### 5.2 Linguistic naturalization

A second domain of passing involves rewriting AI language so that scripts read—and can be delivered—as a recognizable human voice rather than a generic machine register. Participants consistently identified distinctive linguistic “tells”: overly formal wording, templated phrasing, and stiffness that signals non-human authorship. S15 offered concrete examples of what must be changed:

“*AI always generates ‘understand' for me, but normally you'd change it to ‘I get' or ‘I got it'… AI uses those -tion ending words; you definitely have to change them to more colloquial language*.”

S13 similarly complained that AI's modifiers “*feel like they were written by a robot—too much like a school essay, with overly fancy rhetoric*.”

Yet creators did not treat the problem as mere formality. They described a deeper absence of vitality and idiosyncrasy that makes language feel uninhabited. S16 captured this deficit:

“*What AI writes lacks spirit and has no distinctive characteristics. I have to add and revise extensively. This part can be quite frustrating and exhausting—it feels like being a teacher who finds a student's writing inadequate*.”

She noted that AI can swing between “overinterpreting” prompts into grandiose sentimentality and producing drafts that are “*insufficient, lacking a bit of vitality*.” Linguistic naturalization therefore requires adding rhythm, specificity, and minor imperfections that signal a particular person speaking.

Some participants attempted to shorten this labor through style imitation. S16 described giving AI an article she had written and asking it to reproduce her style: “*It will analyze my style and output accordingly*.” Yet the “signature” still demanded intervention: “*I see which parts don't sound like what I would say, delete them, then add my own content*.” For platform-native genres that rely on conversational intimacy, creators also adjusted scripts to sound speakable and socially situated. S9 described adding emojis and modal particles so the text “*sounds like something I said, not an official document*.” These edits are invisible when successful, but they are pivotal for passing because they align the output with the creator's established persona—one of the cues through which audiences infer sincerity and authorship.

### 5.3 Narrative restructuring

A third domain of invisible craft concerns narrative structure. Even when individual sentences are rewritten, creators described AI outputs as organisationally formulaic and brand-hostile: the problem is not only “how it sounds” but “how it thinks.” S12 noted that

“*many times the results AI gives are quite formulaic. They can't make your video have a unique personal touch… AI's way of thinking is browsing the internet then summarizing, so it's more like imposing successful cases on you rather than helping you build a personal brand*.”

Passing, in this sense, requires breaking the template: inserting personal episodes, rearranging segments, and making sequencing choices that reflect individual judgment rather than generic summarisation.

S11 offered a vivid account of this reconstruction process. She first constrains the model's output, “*describe this in a paragraph, within a few hundred words*,” to prevent rambling and force condensation. She then treats the result as a quarry rather than a script:

“*After it condenses, I pick out some usable phrases from it. So I'm basically sifting through garbage to find something to use; I won't just repeat exactly what it generates*.”

The metaphor of “garbage sifting” highlights a recurring practice across interviews: creators mine fragments (phrases, metaphors, transitions) and then rebuild the narrative arc around their own experiences and evaluative stance. Through restructuring, AI drafts can be made to pass as individually authored content rather than template-driven aggregation.

### 5.4 Performative embodiment

Finally, creators described a performative layer of passing in which authenticity is produced through embodied delivery. Even carefully rewritten scripts can still appear rigid or mechanical when read on camera, making performance a necessary site of “humanization.” S16 described integrating AI scripts with teleprompter use:

“*Since AI generates the script, I may use a teleprompter and read it in real time while recording. During this process, the script may be rigid and mechanical, so I add impromptu parts. It's like giving a presentation in college: when I look at the PPT content, I definitely add my own impromptu expressions and never just read it word for word*.”

She framed short videos as “*essentially a live, brief performance*,” arguing that audiences should perceive more than recitation:

“*You need to let audiences see more than just you reading, because otherwise, why wouldn't they just read the text themselves? Why watch a mechanical recitation? Therefore, you must add performative elements: tone, emphasis, strategic pauses, and phrases that resonate. All of these require deliberate performance*.”

Creators thus use delivery to counteract rigidity while remaining attentive to credibility risks. Performative embodiment functions as the final gate of passing: it stages spontaneity and presence so that even AI-assisted scripts can be experienced as a human encounter. Taken together, verification, linguistic naturalization, narrative restructuring, and performative embodiment operate as a single passing pipeline. Creators stabilize credibility, align voice, rebuild structure, and then deliver content in ways that produce liveness. The labor remains largely invisible: when passing succeeds, audiences see only “natural” videos, not the hours spent checking, rewriting, and re-performing AI-enhanced drafts.

## 6 Unequal passing capacities and trust-based inequality

The invisible craft labor required for AI passing, which includes epistemic verification, linguistic and narrative humanization, and embodied performance, is not equally accessible to all creators. Instead, passing capacity stratifies along three intersecting dimensions: educational and professional capital, economic resources and team support, and platform position and competitive pressures. These stratifications mean that while access to AI tools is widespread, the ability to transform AI outputs into trustworthy “authentic” content remains highly unequal.

### 6.1 Educational and professional capital

Educational background and professional training fundamentally shape creators' ability to perform epistemic and linguistic work. Creators with advanced degrees and specialized training described systematic approaches to verification and revision that others lacked. Professional competence and domain-specific expertise further differentiate creators' capacities. S16 positioned herself as a teacher evaluating AI's drafts:

“*I often play the role of a teacher, reviewing what it presents. You of course need solid professional competence and knowledge reserves to see where AI is talking nonsense, to identify errors and problems, to correct mistakes. If you can't do this, you can't produce work that moves audiences, quality work, work that stands out*.”

Her “teacher correcting student work” metaphor encapsulates evaluative craft grounded in years of practice.

Undergraduate disciplinary training provided targeted advantages, particularly in language-intensive fields. S15, with language training, stressed heightened sensitivity to register and tone:

“*Because of my language training, I can immediately hear when something sounds off—when formality doesn't match context, when word choice is too literary for casual speech. AI always generates ‘understand'; normally you'd change it to ‘I get' or ‘I got it.' AI uses those -tion ending words; you definitely have to change them to more colloquial language*.”

Such metalinguistic awareness allows these creators to systematically identify and correct AI's “voice,” whereas others spoke more vaguely about “feeling something is weird” without knowing exactly how to fix it.

Creators lacking comparable capital described more precarious strategies. S11 spoke of “*sifting through garbage to find something usable*,” reflecting the absence of structured evaluation criteria. She explained: “*I think the time saved by AI is probably offset by the time I spend screening the ideas*.” Without systematic training in verification or linguistic analysis, these creators invest comparable or greater time in post-generation labor while achieving less polished results.

### 6.2 Economic resources and team support

Economic resources and team support further shape whether passing labor is sustainable. Cost barriers constrain tool access and workflow sustainability. S15 highlighted:

“*Some AI isn't completely free, which is quite deadly. As a blogger I haven't earned much money, and I have to spend another sum on it—I don't think it's worth it*.”

Time scarcity forces creators balancing content production with other employment to make harsh trade-offs between thorough verification, quality refinement, and maintaining update frequency.

Team-based production introduces the starkest divide. S16 contrasted her individual practice with established influencers:

“*Big influencers' teams are more mature. They may have dedicated groups for copywriting and script generation. The whole process is more streamlined and standardized. They have more time and energy to refine every detail. We, as relatively small-scale creators, need to control the time for each link*.”

Large teams can distribute this work and sustain more standardized, iterative refinement than solo creators who must perform all roles themselves.

### 6.3 Platform position and competitive pressures

Platform position—followers, reputation, and perceived influence—shapes passing strategies by altering both the risks of disclosure and the competitive stakes of adoption. S12 articulated asymmetrical expectations:

“*For small-scale creators like us, using AI might help achieve some success by following others' paths. But for top influencers, since they have considerable online influence, if they still use AI for creation, it might truly mislead others*.”

Larger creators face higher authenticity expectations yet may also possess thicker trust buffers that can absorb experimentation.

For smaller creators, disclosure can be reputationally catastrophic. S16 conveyed the existential anxiety of marginal creators:

“*If I were to disclose to my audience that I used AI, I would still feel this is very embarrassing, or rather a very difficult thing to accept, whether for me or for the audience*.”

Without accumulated trust reserves, a single revelation of AI reliance can trigger unfollows or brand withdrawal. She emphasized:

“*I think this matter will actually affect my audience's trust in my content and their recognition of my account's overall image. It will probably lead to more negative opinions*.”

Competitive dynamics further intensify concealment. S16 observed that creators who refuse AI “will be at a greater disadvantage” because “*they don't know how to use AI to maximize their advantages and promote themselves, nor do they know how to go with the flow*.” Yet she also articulated the moral cost: “*Using AI feels like I'm compromising with this homogeneous market, and even giving up part of my uniqueness. I struggled with this for a while*.” AI adopters may gain speed and volume advantages while masking their tools; non-users sacrifice efficiency for authenticity that is increasingly invisible and undervalued. Only some adopters, however, can sustain the verification and humanization work needed to make AI use both safe and effective under platform scrutiny.

Together, these stratifications produce what we term trust-based inequality: systematic disparities in creators' capacities to maintain audience, client, and platform trust while using AI. High-capital creators—equipped with educational and professional competence, economic resources, and secure platform status—can invisibly leverage AI to scale production while preserving or even enhancing their authenticity persona. Low-capital creators face harsher trade-offs: invest unsustainable labor in passing, accept quality or trust risks, or fall behind in an increasingly AI-accelerated competition.

## 7 Discussion and conclusion

Generative AI has rapidly entered China's short-video creator economy, becoming a practical resource for ideation, scripting, and copywriting under intense pressures to post frequently and remain competitive. This article shows that AI assistance does not simply reduce labor. In authenticity-oriented environments where creators are expected to appear personal, spontaneous, and credible, visible AI involvement can become a reputational liability. Creators therefore face a legitimacy problem that is simultaneously epistemic, relational, and institutional. They must maintain credibility, protect persona-based trust, and navigate platform governance while using tools that audiences may treat as discrediting mediators.

### 7.1 AI passing as sociotechnical repair

This article introduced AI passing as the strategic effort to conceal and humanize AI-assisted content so that it plausibly passes as fully human-authored under platform authenticity expectations. Building from Goffman (1963) notion of passing as the management of potentially discreditable information, AI passing is conceptualized here as a sociotechnical practice rather than a stable identity trait or a moral claim about creators' intentions. This framing extends creator economy scholarship that treats authenticity as labor and craft rather than essence (Abidin, 2016, 2021; Duffy, 2017). When generative AI enters creator workflows, it introduces a new mediator into an already moralized economy of realness, and thereby intensifies the need to manage legibility.

Empirically, AI passing is sustained through recurring forms of invisible authenticity labor that convert AI-assisted drafts into content that can be received as credible and persona-consistent. These practices are repair-intensive by design. When they succeed, both the AI's role and the human work of domestication disappear behind the surface of “natural” content. This aligns with scholarship on invisible work and repair, which shows that essential labor becomes least visible when systems function smoothly (Star and Strauss, 1999) and that coordination and adjustment are the connective tissue of practice (Strauss, 1985). In this sense, what appears as automation is frequently enabled by human repair and maintenance rather than seamless substitution (Graham and Thrift, 2007; Jackson, 2014).

Situating AI passing within this framework connects it to a broader pattern in platformed cultural production. Each time a new productive technology enters creative workflows, whether recommendation algorithms, editing tools, or now generative AI, it can destabilize established norms of authorship and effort, prompting practitioners to develop new repertoires of legitimacy management (Abidin, 2016; Duffy, 2017). What distinguishes the present moment is the specific convergence of unprecedented speed of AI adoption, platform-level algorithmic governance that can detect and penalize AI traces, and the centrality of persona-based trust to creator livelihoods. AI passing is therefore not an aberration unique to generative AI but an intensified instance of a recurring dynamic in which technological mediation must be strategically obscured to preserve authenticity.

### 7.2 Disclosure as conditional trust work

The findings contribute to debates on conditional trust in algorithmic mediation. Prior research warns against assuming that visible AI use uniformly undermines credibility (Dietvorst et al., 2015; Logg et al., 2019; Jago and Carroll, 2023). Our interviews reveal a contrasting pattern: in China's authenticity-oriented short-video production, creators often treat disclosure as carrying uncertain and potentially catastrophic reputational costs, and consequently invest in making AI involvement socially ignorable. This highlights how evaluation is organized through authenticity regimes in which credibility is anchored in experiential plausibility and personal voice. Disclosure is further interpreted through local expectations about platform power, monetisation, and manipulation, which shape whether openness is read as accountability or as strategic performance (Pan and Liu, 2025; Lu, 2025).

The regime-dependent character of AI passing becomes analytically sharper when contrasted with the phenomenon of “AI slop”—a term increasingly used to describe the flood of low-quality, minimally curated AI-generated content that accumulates on platforms where algorithmic recommendation rewards volume over perceived authenticity. In such environments, AI-generated material circulates openly and the central problem is oversaturation rather than concealment. AI passing, by contrast, emerges specifically where authenticity regimes are robust enough that AI involvement functions as a discrediting attribute rather than a normalized production input. This contrast clarifies that AI passing is not a universal response to generative AI adoption but a situated practice contingent on how platforms, audiences, and genre norms co-produce the conditions under which AI mediation is evaluated. The deviant case of S3 offers empirical grounding for this claim: in his utilitarian travel-guide niche, audiences evaluated output rather than process, enabling him to reframe disclosure as a persona asset rather than a reputational liability. His case confirms that the strength of authenticity regimes, and therefore the necessity of AI passing, varies across content genres within the same platform ecosystem.

### 7.3 AI assistance as invisible repair labor

A key implication of AI passing is an efficiency paradox. AI can accelerate drafting, but creators' workloads are frequently relocated into downstream repair, including verification, re-voicing, restructuring, and re-performing. Rather than replacing human expertise, generative AI reorganizes it into less visible and often more skill-intensive forms. This pattern matches classic accounts of how automation shifts work into articulation, maintenance, and coordination rather than eliminating it (Strauss, 1985; Star and Strauss, 1999; Jackson, 2014). In authenticity-centered creator economies, this shift is especially consequential because the most valued aspects of content, including voice, intimacy, and liveness, are precisely the qualities creators must reinstall through repair and performance.

This also clarifies why the labor of AI-assisted creation is systematically undervalued. Successful passing is designed to leave no trace. Audiences encounter smooth, “human” content rather than the friction that produced it, and platforms and tool imaginaries can normalize a narrative in which AI reduces effort even when creators describe the opposite. When passing fails, however, creators bear reputational penalties while the labor itself remains largely unrecognized. AI passing thus converts uncertainty about AI-generated content into intensified behind-the-scenes labor that is difficult to make visible as work.

### 7.4 AI passing and trust-based inequality

Because AI passing is repair-intensive craft labor, it is not equally accessible. Passing requires epistemic judgement, metalinguistic control, genre competence, and performative skill, as well as time and sometimes team support to iterate and refine. These requirements intersect with educational and professional capital, economic resources, and platform position. The result is trust-based inequality, defined here as systematic disparities in creators' capacity to maintain epistemic, relational, and institutional trust while using AI. Generative AI therefore risks functioning less as a democratizing technology than as an inequality-amplifying infrastructure.

These dynamics carry implications for platform governance and creator-tool relations. Detection and labeling regimes often presume a binary distinction between human and AI authorship, yet our data indicate continuum practices in which creators may rely on AI at the point of generation but invest substantial labor in verification, rewriting, restructuring, and performance. Binary framings risk erasing craft labor and may stigmatize careful collaboration while rewarding more sophisticated concealment. Supporting healthier AI-mediated creator ecologies therefore requires recognizing that credibility is produced through repair-intensive practices and that governance choices can amplify or mitigate inequalities in who can afford those practices.

This study is qualitative and mechanism-oriented, with strong representation from Xiaohongshu and a focus on text-based AI use in scripting and copywriting. Future research could compare platforms and genres more systematically, examine audience interpretations of AI traces and disclosure cues, and quantify how repair labor varies by creator resources, follower tiers, and production organization. The deviant case of S3 further suggests that disclosure dynamics may vary substantially across content genres, warranting investigation with more genre-diverse samples. Beyond the creator economy, the concept of AI passing has analytical relevance for other professional contexts where generative AI is increasingly adopted but norms of individual authorship and expertise remain strongly enforced. DeJordy (2008) generalization of passing as a structural feature of any situation involving invisible, potentially discreditable attributes already authorizes this conceptual migration. Academic production, journalism, and legal drafting each involve practitioners balancing efficiency gains against professional norms that valorise human judgement—precisely the conditions under which repair-intensive concealment becomes a pragmatic response. Extending the AI passing framework to these domains would test its analytical portability and clarify whether the dynamics documented here are specific to creator economies or characteristic of AI-mediated knowledge work more broadly.

Ultimately, the scripts that appear in creators' short videos are shaped by platform and generative AI mediation, but they are equally forged through creators' creative labor and ongoing negotiations of agency. What audiences perceive as liveness is an achieved effect that creators actively install through checking, re-voicing, restructuring, and re-performing AI-assisted drafts. At a deeper level, while AI promises efficiency at the point of generation, it can quietly challenge creators' control over their own creative process by shifting work downstream into repair and performance. In doing so, it produces an often invisible redistribution of authorship and subjectivity at the heart of platformed cultural production.

## Data Availability

The raw data supporting the conclusions of this article will be made available by the authors, without undue reservation.
